# Micronutrients-fortified rapeseed oil improves hepatic lipid accumulation and oxidative stress in rats fed a high-fat diet

**DOI:** 10.1186/1476-511X-12-28

**Published:** 2013-03-06

**Authors:** Jiqu Xu, Xiaoqi Zhou, Hui Gao, Chang Chen, Qianchun Deng, Qingde Huang, Jing Ma, Zhengyang Wan, Jin’e Yang, Fenghong Huang

**Affiliations:** 1 Department of Product Processing and Nutriology, Oil Crops Research Institute, Chinese Academy of Agricultural Sciences, 2 Xudong Second Road, , Wuhan 430062, People’s Republic of China; 2 Hubei Key Laboratory of Lipid Chemistry and Nutrition, 2 Xudong Second Road, , Wuhan 430062, People’s Republic of China; 3 Department of Nutrition and Food Hygiene, School of Public Health, Tongji Medical College, Huazhong University of Science and Technology, 13 Hangkong Road, Wuhan 430030, People’s Republic of China; 4 Department of Gastroenterology, The No.1 Hospital of Yichang, 2 Jiefang Road, Yichang 443000, People’s Republic of China; 5 Yichang Center for Disease Control and Prevention, 3 Dalian Road, Yichang 443000, People’s Republic of China

**Keywords:** Rapeseed oil, Polyphenols, Tocopherols, Phytosterols, Lipid accumulation, Oxidant stress

## Abstract

Intake of high-fat diet is associated with increased fatty livers. Hepatic lipid accumulation and oxidative stress are key pathophysiological mechanisms in this disease. Micronutrients polyphenols, tocopherols and phytosterols in rapeseed exert potential benefit to hepatoprotection, but most of these micronutrients are removed by the traditional refining process. The purpose of the present study was to determine whether rapeseed oil fortified with these micronutrients can decrease hepatic lipid accumulation and oxidative stress induced by high-fat diet. Sprague–Dawley rats received rodent diet contained 20% fat whose source was refined rapeseed oil (RRO) or fortified RRO with low, middle and high quantities of these micronutrients for 10 weeks. Intake of RRO caused a remarkable hepatic steatosis. Micronutrients supplementation was effective in reducing steatosis as well as total triglyceride and total cholesterol contents in liver. These micronutrients also significantly increased hepatic antioxidant defense capacities, as evaluated by the significant elevation in the activities of SOD and GPx as well as the level of GSH, and the significant decline in lipid peroxidation. These findings suggest that rapeseed oil fortified with micronutrients polyphenols, tocopherols and phytosterols may contribute to prevent fatty livers such as nonalcoholic fatty liver disease by ameliorating hepatic lipid accumulation and oxidative stress.

## Introduction

The typical diet in modern industrialized societies is high-fat content. Chronic consumption of this type of diet is considered a major cause of a variety of health problems including obesity, diabetes and cardiovascular disease [1]. It is well known that liver is the main organ for lipid metabolism and its lipid composition and content depend on the diet. In fact, feeding a high-fat diet for long periods of time can have adverse effects on liver and result in occurrence of nonalcoholic fatty liver disease (NAFLD) which is one of the leading causes of hepatic dysfunction in the modern world and includes a broad spectrum ranging from benign hepatic steatosis to cirrhosis [2]. Regardless of etiology of NAFLD, lipid accumulation and oxidative stress are two requisite for this disease progression [3].

Rapeseed oil is one of the most common edible oils in the world. It possesses an exceptionally low amount of saturated fatty acids and the predominantly of monounsaturated fatty acids as well as a well-balanced ratio between α-linolenic acid and linoleic acid. The composition of fatty acid in rapeseed oil can induce many responses in carbohydrate metabolism, inflammatory cytokines and adipose tissue [4], which make rapeseed oil of benefit to health. For example, replacing dairy fat with rapeseed oil can cause a rapid and clinically relevant improvement in serum lipoprotein profile including lowering of triglycerides in hyperlipidaemic individuals [5].

In addition to triacylglycerols, there are many micronutrients such as phenolic compounds, phytosterols and tocopherols presented in rapeseed oil. These micronutrients are naturally abundant in rapeseed seed and have been reported to possess various health benefits. For example, these micronutrients exhibit excellent antioxidant activity which have been attributed to their redox properties and may function as free radical scavengers or potential chelators of prooxidant metals. Phytosterols can decrease hepatic cholesterol concentrations by inhibiting cholesterol absorption [6]. Besides, it has been demonstrated that phenolics have the ability to ameliorate hepatic lipid accumulation in rodent fed a high-fat diet [7]. All these beneficial effects of these micronutrients might play important roles in preventing the initiation and development of fatty livers. However, most of these micronutrients are removed in traditional processing technology currently used in the world (extraction and refining), which will have an adverse effect on the hepatoprotective effect.

In order to increase the levels of these micronutrients in rapeseed oil, extensive studies of processing technologies have been done. However, it is difficult to produce high-quality virgin rapeseed oil until now [8]. Therefore, artificially adding these micronutrients to refined oil may be another simple and expedient method. In our previous report [9], we have demonstrated that rapeseed oil fortified with micronutrients (polyphenols, tocopherols and phytosterols) exerts a cardiovascular protective effect. The purpose of the present study was to determine whether this kind of fortified refined rapeseed oil is able to decrease hepatic lipid accumulation and oxidative stress in rats fed a high-fat diet.

## Materials and methods

### Fortification of rapeseed oil with micronutrients

The refined low erucic acid rapeseed oil was purchased from Hulunbuir Jinjiao Bio-chemical Ltd (Inner Mongolia, China), and its levels of tocopherols, phytosterols and phenolic compounds have been analyzed and shown in our previous study [9]. Plant sterol esters (Vegapure 95E®, Cognis GmbH, Germany), fat-soluble tea polyphenols (Pulimeidi biotech, China), and tocopherols (Wuhan Yuancheng, China) were used as the fortificants in the present study. The rapeseed oil was fortified with three doses of these fortificants and the contents of these micronutrients in the fortified oil were also shown in our previous study [9].

### Animals and diets

Forty male Sprague–Dawley rats (Sino-British Sippr/BKShanghai, China), initially weighing 150–170 g, were used in this study. The rats were housed individually under conditions of constant temperature (24 ± 1°C) and a standard dark cycle (20.00–08.00) with access to laboratory chow and tap water ad libitum. After a 1-week acclimation period, the animals were randomly divided into four groups of 10 animals each: the refined rapeseed oil (RRO) group, fortified refined rapeseed oil with low, middle and high contents of these fortificants (L-, M-, and H-FRRO) groups. The high-fat diet contained 35% maize starch, 20% casein, 15% glucose, 5% cellulose, 3.5% mineral mixture (AIN-93 M), 1% vitamin mixture (AIN-93 M), 0.2% choline bitartrate, 0.3% DL-methionine and 20% fat. The fat in the diet was provided by different rapeseed oils mentioned above. The animals were cared for in accordance with *the Guiding Principles in the Care and Use of Animals*. The experiment was approved by the Oil Crops Research Institute Council on Animal Care Committee, Chinese Academy of Agricultural Sciences.

### Tissue preparation

After 10 weeks of treatment, rats were fasted for 16 hours and then killed under anaesthesia. Liver was rapidly dissected, weighed, and a small piece of right liver lobe was fixed in 4% paraformaldehyde for hematoxylin-eosine staining. The remaining liver tissue was stored at -80°C until analysis.

### Assay of liver lipid content

Lipids were extracted from 1 g liver with a mixture of chloroform/methanol (2:1, v/v) by the method of Folch [10]. Total triglyceride (TG) and total cholesterol (TC) contents were measured with commercial kits (Zhongsheng Beikong Biotech Company, China).

### Assay of liver antioxidant capacity and lipid peroxidation

Superoxide dismutases (SOD) activity was measured according to the method of Kono [11]. Catalase (CAT) activity was estimated basing on the method of Goth [12]. Glutathione peroxidase (GPx) activity was measured by the method of Sazuka [13]. The glutathione (GSH) content was determined by the method of Moron [14]. Thiobarbituric acid reactive substances (TBARS) level was estimated by the method of Buege and Aust [15].

### Assay of protein concentration

The protein concentration was determined according to the method of Lowry [16], using bovine serum albumin (BSA) as standard.

### Statistical analyses

Results were expressed as mean ± SEM (standard error of the mean). Statistical analysis were based on one-way ANOVA, followed by the Fisher PLSD post hoc test if the overall differences were significant. All statistical analyses were performed using SPSS 13.0 statistical software (SPSS Inc., Chicago, IL) and the limit of statistical significance was set at *p* < 0.05.

## Results

### Liver histology

**Figure 1:**
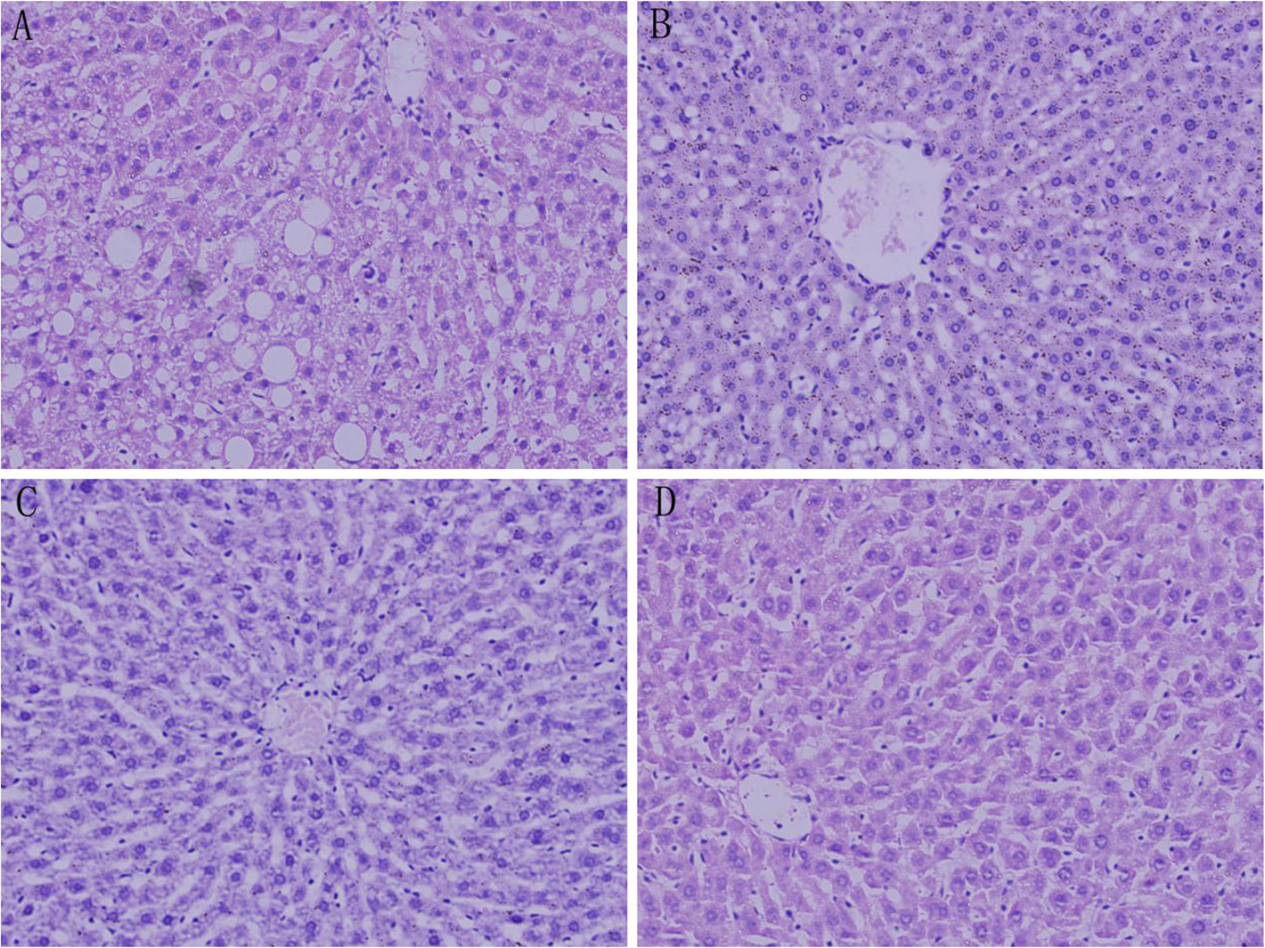
Liver histology after hematoxylin-eosine staining of liver sections from a representative rat from each group: (A) the refined rapeseed oil group, (B) fortified refined rapeseed oil with low contents of micronutrients group, (C) fortified refined rapeseed oil with middle contents of micronutrients group and (D) fortified refined rapeseed oil with high contents of micronutrients group.

As shown in Figure 1, extensive microvesicular steatosis and scattered foci of macrovesicular steatosis were observed in the liver sections of rats fed with the RRO for 10 weeks. A marked reduction in circular lipid droplets in both number and size was noted in the livers from rats treated with L- FRRO, and the liver in M-, and H-FRRO groups appeared minimal and almost normal fat deposition in hepatocytes. In addition, there was no evidence of unequivocal inflammation in all groups.

### Liver lipids content

**Figure 2:**
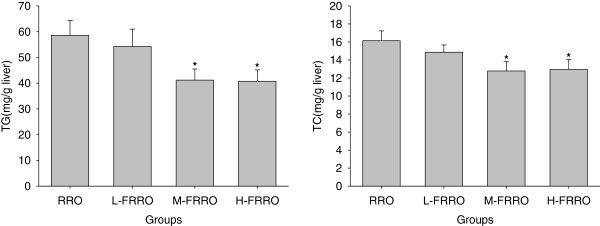
**Effects of rapeseed oil fortified with micronutrients (polyphenols, tocopherols and phytosterols) on hepatic TG and TC contents of rats fed a high-fat diet.** RRO: the refined rapeseed oil group; L-. M- and H- FRRO: fortified refined rapeseed oil with low, middle and high contents of micronutrients groups. Bars represent the mean ± SEM from 10 animals in each group. * *p* < 0.05 compared to the RRO group.

Consistent with histologic assessment, and as can be seen from Figure 2, although L-PRRO administration only had a trend to down-regulate both hepatic TC and TG, the reduction was significant in animals treated with M- and H-PRRO when compared with rats that received RRO.

### Liver antioxidative capacity and lipid peroxidation

**Figure 3:**
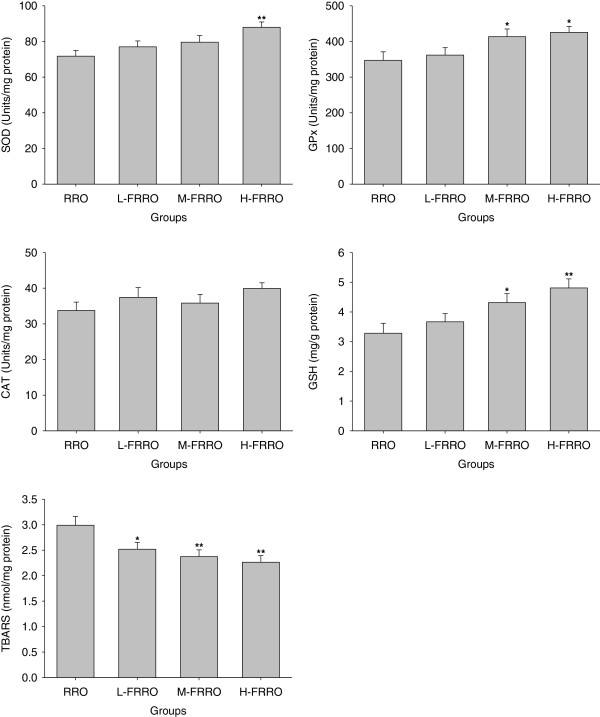
**Effects of rapeseed oil fortified with micronutrients (polyphenols, tocopherols and phytosterols) on the activities of antioxidant enzymes (SOD, GPx and CAT), the levels of GSH and the contents of TBARS in liver of rats fed a high-fat diet.** RRO: the refined rapeseed oil group; L-. M- and H- FRRO: fortified refined rapeseed oil with low, middle and high contents of micronutrients groups. Bars represent the mean ± SEM from 10 animals in each group. * *p* < 0.05 and ** *p* < 0.01 compared to the RRO group.

As shown in Figure 3, there were not significant changes in hepatic CAT activities in all the experimental groups. SOD activity in H-FRRO group and GPx activities in M-, and H-FRRO groups were significantly higher than that of RRO group. In addition, when compared with animals administrated with RRO, the hepatic levels of GSH in rats of M-, and H-FRRO groups were also significantly elevated. Rats in all FRRO groups revealed markedly lower TBARS contents in liver than animals treated with RRO.

## Discussion

Rapeseed oil possesses an optimum fatty acid composition, which makes this oil exert a potentially positive health effect in many aspects [5,17,18]. But unfortunately, rapeseed oil may cause significant lipid accumulation in liver even though the diets were not high in fat, and this adverse effect is similar to that of lard [19]. This fact increases the concerning about the detrimental effects on liver along with rapeseed oil consumption. In addition to triacylglycerols, rapeseed also contains many kinds of micronutrients which have excellent bioactivity. These micronutrients include phytosterols and various antioxidants such as polyphenols, tocopherols and coenzyme Q. All these bioactive molecules work in concert and may contribute to prevent the adverse effects induced by high-fat diet. But unfortunately, most of these micronutrients are lost during the oilseed oil refining. Therefore, artificially adding micronutrients (polyphenols, tocopherols and phytosterols) to refined rapeseed oil may have a beneficial effect on hepatoprotection.

In the present study, as was expected, RRO-rich diet resulted in remarkable hepatic steatosis, which meant the pronounced fat accumulation. FRRO, especially M-, and H-FRRO, marked ameliorated the severe steatosis in liver. Accordingly, M-, and H-FRRO declined both hepatic TG and TC significantly. The micronutrients phytosterols, polyphenols and tocopherols were all responsible for these beneficial changes. Catechins, the major polyphenols found in green tea, have been reported to have ability to form complexes with lipids and lipolytic enzymes, and thereby interfering with the luminal processes of emulsification, hydrolysis, micellar solubilization, and subsequent uptake of lipids [20]. As a result, catechins lower the intestinal absorption of lipids effectively [20,21], and which may partly contribute to the decline of hepatic lipid accumulation. Furthermore, catechins can be absorbed and detected in high levels in liver [22]. It has been reported that catechins are able to decrease hepatic lipogenesis by regulating different enzyme activities. In addition to acting as a natural inhibitor of fatty acid synthase [23,24], polyphenols also reduce the expression of stearoyl-CoA desaturase-1 [21,24] which is the rate-limiting enzyme in the synthesis of monounsaturated fatty acids in liver, malic enzyme [21] which involves in lipid synthesis, and 3-hydroxy-3-methyl-glutaryl-CoA reductase (HMGR) [24] which is the rate-limiting enzyme for cholesterogenesis. On the other hand, catechins upregulate the expression of acyl-CoA oxidase and medium chain acyl-CoA dehydrogenase, which are key enzymes for the β-oxidation of fatty acids, and hence stimulate hepatic lipid catabolism [25]. Phytosterols are structurally similar to cholesterol but themselves are absorbed only in trace amounts [6], and for this reason, these compounds inhibit cholesterol absorption including recirculating endogenous biliary cholesterol which is a key step in cholesterol elimination [6]. These meant that although there was little cholesterol contained in the rodent diet in this study, inhibition of intestinal cholesterol absorption was still the main mechanism responsible for the cholesterol-lowering effect of phytosterols. Therefore, although supplement of phytosterols increases the hepatic concentrations of these compounds [26,27], which increase the activity of HMGR [28], these compounds still reduce hepatic TC significantly [26,27]. In addition, despite increases in hepatic lipogenic gene expression and de novo lipogenesis, phytosterols are found to reduce hepatic triglyceride concentrations pronouncedly by increased fecal fatty acid loss [26]. Another micronutrient tocopherols have also been reported to slash hepatic triglyceride content in a model of NASH induced by high-fat diet [29], although the precise mechanism remains to be defined.

High-fat diet reduces the levels of hepatic antioxidants, which induces oxidative stress remarkably [29,30]. Polyphenols act as antioxidants through not only scavenging reactive oxygen and chelating redox-active transition metal ions directly, but also inhibition of prooxidant enzymes and induction of antioxidant enzymes indirectly [31]. For example, polyphenols inhibit the activities of inducible nitric oxide synthase, cyclooxygenases, lipoxygenases and xanthine oxidase but induce the activities of CAT, GPx and SOD [31]. Besides, polyphenols possess the property of increasing GSH concentrations in tissues [31]. As a kind of potent lipid-soluble antioxidants, tocopherols are also effective against liver lipoperoxidation induced by high-fat diet [29]. Beyond cholesterol-lowering effects of phytosterols, these compounds also upregulate GSH level and some antioxidant enzymes activities such as Mn-SOD and GPx by the estrogen/phosphatidylinositol 3-kinase pathway [32]. The joint actions of all these antioxidants are able to increase antioxidant capacity higher than that provided by each separate compound [33-35]. In parallel with the enhancement of these micronutrients in the present study, the activities of SOD and GPx as well as GSH level in liver elevated significantly. The increased antioxidant capability in liver combining with the remarkable decline of hepatic TBARS levels in all doses of FRRO suggested that FRRO were favorable to attenuate oxidative stress and therefore prevent the progression of fatty livers. Agree with these findings, optimized rapeseed oils which contain naturally abundant these micronutrients have been reported to increase antioxidant status and reduce lipid peroxidation in liver as well as in plasma and brain [36,37].

In summary, rapeseed oil fortified with tocopherols, polyphenols and phytosterols has the ability to reduce excessive hepatic fat accumulation and oxidative stress. These results indicated that the rapeseed oil fortified with these micronutrients might contribute to ameliorate fatty livers (such as NAFLD) induced by high-fat diet.

## Competing interest

No competing financial interests exist.

## Authors’ contributions

JX designed and wrote a first draft of the paper. XZ, HG, QD, JM, ZW and JY carried out all the experiments. CC and QH performed the data analysis and created the figures. FH contributed to the design of the study, reviewed the manuscript and contributed to the final version. All authors contributed to and have approved the final manuscript.
